# Of Creatures Great and Small: The Advantages of Farm Animal Models in Immunology Research

**DOI:** 10.3389/fimmu.2013.00124

**Published:** 2013-05-27

**Authors:** Amanda J. Gibson, Tracey J. Coffey, Dirk Werling

**Affiliations:** ^1^Department of Pathology and Infectious Diseases, Royal Veterinary CollegeLondon, UK; ^2^School of Veterinary Medicine and Sciences, University of NottinghamSutton Bonington, UK

In recent decades the inappropriate use of antibiotics in farm animals, either as growth enhancers or for treatment of infectious diseases, has resulted both in higher concentrations of antibiotic residues in meat destined for the human food chain and in an increasing prevalence of antibiotic-resistant bacteria (Carlet et al., 2012). Increased public concerns regarding their potential impact on human health may result in a reduction or cessation in the use of antibiotics in farm animals (Veterinary Record, 2011, 2012). Thus, alternative treatments for the control of infectious diseases in farm animals need to be identified as a research priority. In achieving this goal, the physiological relevance of an animal model to the intended target population becomes a key factor. The majority of models currently used for pharmaceutical research are murine and it is possible that potentially valuable therapeutics are discounted on the basis of lack of responses generated in this system, as shown very recently for sepsis (Seok et al., 2013). Several non-rodent species, such as cattle, pigs, and chicken, are both valuable models for infectious diseases in humans (Hein and Griebel, 2003; Visscher and Goddard, 2011; Waters et al., 2011; Costa et al., 2012; Meurens et al., 2012) and important clinical targets in their own right. Effective vaccines have been developed for many acute viral and bacterial infections, whereas for others, like tuberculosis (TB), there is still a need for reliable vaccines. Pathology seen in the murine models of TB currently used is very different to the pathology seen in humans (Gupta and Katoch, 2009), whereas the bovine and human diseases share many similarities (Waters et al., 2011). For such diseases it is crucial that we use natural disease-models, with the appropriate pathogen in the most appropriate host, to examine the host-pathogen interactions that occur in outbred populations. A fundamental point in the development of new and improved intervention strategies is the understanding of host differences in the innate immune response, which primes the subsequent adaptive immune response. Although the innate immune system has been largely conserved during evolution, marked variations and diversity exist between different mammalian species within Pattern-Recognition-Receptor (PRR) structure (Jungi et al., 2011). These differences are based on evolutionary pressure within the innate immune system, potentially reflecting the specific threats encountered by each species (Zhang et al., 2010). This selective pressure appears to be absent in the available murine sequences (Werling et al., 2009). The similarity between human and farm animal PRR (Jungi et al., 2011) is further supported by their similar response to ligands (Kapetanovic et al., 2012), in contrast to murine PRR (Hajjar et al., 2002; Grabiec et al., 2004; Farhat et al., 2010). Since recognition by PRR is associated with adaptive immunity by providing optimal immunostimulation, learning more about these key molecules in farm animals might inform us about their adjuvant effect in vaccines for use in these animals as well as humans. Given the size and blood volumes of farm animals, there are also greater opportunities to repeatedly access to different cell types – an asset which would facilitate the assessment of cell specific effects of such immunomodulatory agents on autologous cells (Hein and Griebel, 2003). Farm animal models do have their disadvantages such as dedicated housing, biosecurity, and the confinement of infected animals. However, it is misleading to rely on murine models to fill the gaps in our knowledge of disease-pathogenesis, and choosing a research model should be more than just a matter of convenience and convention (Bolker, 2012).

However, in addition to their potential use as models for human diseases, research in farm-animals models have a “right-on-their-own,” taken the predicted increase in food supply necessary by 70% to support an ever-increasing global population as well as the occurrence of new emerging diseases, such as Schmallenberg virus into account. These challenges can be faced and overcome by an increased readiness of farm animal research centers to apply their models in novel ways, a willingness of the medical profession to accept more suitable disease-models, and a willingness of funding agencies and companies to invest in this type of research partnership.
